# Social Determinants of Health and Neurocognitive Aging in Indigenous and Non-Indigenous Older Adults in Rural Andean Ecuador: A Population-Based Study in Cotacachi

**DOI:** 10.3390/brainsci16101002

**Published:** 2026-09-22

**Authors:** Kuri Tituaña-Vega, Ivan Palacios-León, Carmen Paredes-Manrique, Jonathan Adrian Zegarra-Valdivia

**Affiliations:** 1 Global Brain Health Institute, University of California, San Francisco, CA 94158, USA; 2 College of Health Sciences, School of Medicine, Universidad San Francisco de Quito, Quito 170901, Ecuador; tpalacios@usfq.edu.ec; 3 Universidad Tecnológica del Perú, Lima 15046, Peru; c27709@utp.edu.pe; 4 Psychology Department, Faculty of Health Sciences, Universidad Científica del Sur, Lima 15067, Peru

**Keywords:** social determinants of health, cognitive aging, dementia, indigenous populations, rural health, educational inequality

## Abstract

**Introduction**: Social determinants of health shape dementia risk in Latin America, particularly in rural and Indigenous populations. **Methods:** This cross-sectional study examined cognitive impairment and associated social factors in 75 adults aged 60 years or older in Cotacachi, Ecuador, identifying as Indigenous, Mestizo, or Other. Cognition was assessed with the RUDAS using education-adjusted cutoffs, alongside daily functioning and depression. **Results:** Cognitive impairment affected 22.7% and was more frequent among Indigenous participants. Limited early education emerged as a key social factor. In adjusted analyses, older age and depression were associated with impairment, whereas vascular conditions were not; ethnicity could not be disentangled from rurality. **Discussion:** These findings suggest that observed ethnic differences are embedded within structural inequalities, particularly educational and territorial disadvantage, supporting dementia prevention strategies addressing social inequities and mental health in rural Andean communities.

## 1. Introduction

Population aging in Latin America is accelerating, with rising prevalence of mild cognitive impairment (MCI) and dementia, particularly among women, rural residents, and people with low education [1]. These trends challenge fragile health systems, long-term care, and family support networks [2,3]. Ecuador reflects this transition: the 2010 National Survey of Health, Well-being, and Aging reported higher cognitive impairment among rural older adults, especially women with fewer than six years of schooling [4].

Among adults older than 60 years, dementia prevalence in Latin America exceeds 8% and is projected to double in coming decades [2,3]. MCI prevalence ranges from 6.8% to 25.5% [1], and dementia prevalence averages 10.7%, with higher rates among women, rural residents, and people with low education [5]. Up to 54% of dementia cases in the region are attributable to modifiable risk factors, including obesity, physical inactivity, depression, hypertension, and diabetes [6], underscoring the potential of prevention.

In the Andean region, where Indigenous populations are concentrated in rural areas, poverty, ethnic discrimination, and fragmented health systems shape life-course exposure and access to care [7,8]. Cotacachi, Ecuador, exemplifies this setting, where structural social determinants, vascular risk accumulation, and cultural diversity converge [9,10]. Rural Indigenous communities continue to face limited access to water, sanitation, transportation, and health facilities [8]. Health-system segmentation further widens inequities in access and quality [7,11]. Geographic distance, lack of trained personnel, poor intercultural adaptation, and out-of-pocket costs delay diagnosis, treatment, and long-term dementia care.

Evidence suggests that dementia risk in Latin America is driven more by social determinants than by genetic ancestry [12]. The “social exposome” concept highlights how cumulative exposure to poverty, educational disadvantage, and environmental stress affects brain health trajectories [13]. Educational deprivation, food insecurity, and social exclusion explain much of the variability in dementia prevalence across Indigenous groups [12,14], while loneliness, depression, social isolation, and vascular disease also predict cognitive decline [15,16]. Some subsistence-based groups such as the Tsimane and Moseten show low dementia prevalence, likely because of protective lifestyles [17], but structurally marginalized Indigenous populations often experience greater risk [5,10].

Within Ecuador, previous studies from rural communities have shown that cognitive aging is associated with combinations of clinical, vascular, and social conditions [9,16]. Cotacachi is particularly informative because Indigenous identity, rural residence, limited early-life educational opportunities, and restricted healthcare access strongly converge within the same geographically defined population. For the generation represented in this study, formal schooling exposure was particularly limited, making early-life educational opportunity an important contextual factor when interpreting late-life cognitive performance.

A major challenge in rural settings is cognitive screening. Common instruments such as the MoCA and MMSE are strongly influenced by culture and education. Although adjusted cutoffs can achieve AUC values above 0.95 for dementia detection [18], the MoCA requires local calibration in rural Ecuador [9,19], and the MMSE is limited by educational bias [20]. Region-specific batteries and functional assessments are therefore needed, especially for MCI detection [18,21]. The Rowland Universal Dementia Assessment Scale (RUDAS) reduces cultural bias and has shown high diagnostic accuracy in low-educated South American populations [18,21,22], particularly when combined with functional measures such as the Pfeffer Functional Activities Questionnaire (PFAQ) [23,24]. Within this framework, this study aimed to characterize the neurocognitive status of adults aged 60 years and older in Cotacachi and quantify exposure to social determinants of health at structural and individual levels.

## 2. Materials and Methods

### 2.1. Study Design and Framework

This community-based cross-sectional study examined neurocognitive status and its association with structural and individual social determinants among adults aged 60 years and older in Cotacachi, Ecuador. It was guided by a life-course framework in which structural disadvantage, poverty, low education, ethnic marginalization, rurality, and limited healthcare access contribute to late-life cognitive vulnerability through structural and intermediary factors including health-system fragmentation, territorial isolation, discrimination, vascular risk, psychosocial stress, depression, and educational exposure. Cotacachi is a rural Andean municipality with a large Indigenous population, marked socioeconomic vulnerability, educational inequities, and limited healthcare infrastructure.

### 2.2. Participants

Seventy-five community-dwelling older adults were enrolled (mean age 72.32 years, SD 6.03; 78.7% female). Participants self-identified as Indigenous (*n* = 35), Mestizo (*n* = 35), or Other (*n* = 5). Eligibility criteria included age ≥ 60 years, birth in Ecuador, residence in the canton of Cotacachi, ability to communicate in Spanish, and availability of a close informant familiar with the participant’s everyday functioning. Written informed consent was obtained from the participant or legal representative, as appropriate, and from the informant. Exclusion criteria included severe visual or auditory impairment that substantially prevented completion of the assessment, previously diagnosed incapacitating or poorly controlled psychiatric illness, and inability to identify an appropriate informant. Pre-existing cognitive impairment was not an exclusion criterion, because the study was designed to characterize the full spectrum of cognitive status in the community. A previous history of stroke or traumatic brain injury was likewise not an automatic exclusion criterion; these conditions were recorded as part of the structured medical and neurological history. The data collection period spanned from September 2022 to February 2024.

### 2.3. Procedures

Trained evaluators applied a standardized dementia protocol in community settings. Assessments followed a fixed sequence: sociodemographic characteristics and early educational exposures; structural social determinants, including healthcare access, insurance, discrimination, and social adversity; clinical history and lifestyle factors, including vascular-metabolic conditions, medication use, and comorbidities; cognitive testing; functional assessment; depressive symptoms; and perceived stress during the prior year. Evaluators received standardized training in administration, interviewing, and cultural adaptation.

Participants were recruited through community-based outreach conducted in collaboration with local health authorities and community leaders rather than through a dementia or memory clinic. Potential participants were informed about the study within community settings and, if interested, were subsequently screened for eligibility and invited to participate.

### 2.4. Instruments

A culturally adapted battery for rural Andean populations was used. Sociodemographic and life-course variables included age, sex, self-identified ethnicity, place of residence, marital and household characteristics, and educational history. Educational exposure was characterized by using school attendance (public, private, or no formal schooling), rural or urban location of primary schooling, completed years of education, educational attainment, literacy level, and perceived quality of education. Clinical history was assessed by structured self-report of previously diagnosed conditions, including hypertension, diabetes, dyslipidemia, ischemic heart disease, arrhythmia, cerebrovascular disease, Parkinson’s disease, previous traumatic brain injury with loss of consciousness, and other neurological or psychiatric conditions. Medication use was assessed through daily medication use, number of medications, and treatment adherence.

Lifestyle factors included physical activity, smoking, alcohol consumption, and nutritional indicators. Physical activity was assessed according to participation, type, frequency, and duration. Smoking history was defined as current or former smoking (ever smoking), with additional information on duration and usual number of cigarettes per day. Alcohol exposure included current or previous consumption, duration, frequency, and reported intoxication. Nutritional indicators included number of daily meals and unintentional weight loss during the previous six months. Healthcare access was assessed by whether participants had needed medical attention during the previous 12 months but had been unable to obtain it, together with reported barriers to care.

Social violence was operationalized as discriminatory mistreatment experienced in public or commercial settings. Participants were asked whether they had received poorer service than other people in restaurants, markets, shops, or similar establishments. Those reporting such experiences additionally indicated their frequency and perceived reason, including nationality or place of origin, ethnicity, language, sex or sexual orientation, age, religion, political ideology, physical appearance, disability, socioeconomic or educational status, occupation, or other reasons. A binary indicator identified exposure to this form of social violence, and a composite score summarized the corresponding questionnaire-derived indicators used in the analyses.

Global cognition was assessed with the RUDAS, a 0–30 scale covering memory, visuospatial orientation, praxis, visuoconstruction, judgment, and verbal fluency. In Peru, RUDAS discriminated normal cognition, MCI, and dementia with an AUC of 0.98; cutoffs of <19 and <23 yielded sensitivities of 95% and 89% and specificities of 97% and 93%, respectively [21]. In rural settings, AUC values for dementia detection ranged from 0.991 to 0.994, with overall accuracy above 90% [21]. Compared with the MMSE, RUDAS is less affected by education [22]. Estimates were contextualized against previous Latin American RUDAS studies. In this study, cognitive classification (normal, MCI, dementia) was based on education-adjusted Peruvian cutoffs, functional assessment, and DSM-5 criteria; cutoffs of <22 for control versus MCI and <18 for MCI versus major neurocognitive disorder were used for the rural illiterate population.

Functional status was assessed with the Pfeffer FAQ, an informant-based measure of instrumental activities of daily living; scores range from 0 to 30 and values ≥ 9 indicate significant impairment. The FAQ has shown high internal consistency in Brazil [23,24]. The Family Questionnaire (FQ) was used as an additional informant-based tool; scores ≥ 3 indicated meaningful decline. Depressive symptoms were assessed with the GDS-15, using thresholds of 0–5 (normal), 6–9 (mild), and ≥10 (clinically significant). Although not validated in Ecuador, the GDS-15 has shown acceptable reliability, sensitivity, and specificity in Latin American populations [25,26].

### 2.5. Preliminary Psychometric Evaluation of RUDAS

Given the documented cross-cultural performance of the RUDAS and its previous validation in low-educated and illiterate South American populations [18,21,22], a preliminary evaluation of its psychometric performance was conducted in the present sample. This analysis was intended to characterize internal consistency and selected construct-related properties within the Cotacachi cohort rather than to constitute a formal validation of the instrument.

Internal consistency was assessed using Cronbach’s alpha and McDonald’s omega, together with domain–total correlations. RUDAS was administered once to each participant. Accordingly, test–retest reliability was not assessed, and no intraclass correlation coefficient (ICC) was included in the final psychometric evaluation. An ICC across the six RUDAS domains was not considered appropriate because these domains represent distinct cognitive components rather than interchangeable ratings or repeated measurements. No additional global cognitive screening instrument was administered for concurrent comparison, as commonly used alternatives such as the MMSE and MoCA are substantially influenced by educational and cultural factors and were considered less suitable for this low-educated rural setting.

We explored construct-related associations through correlations with demographic, clinical, functional, and psychosocial variables. Given the sample size, these analyses were interpreted descriptively, and the resulting reliability estimates were additionally contextualized against those reported in previous Latin American RUDAS studies, including larger low-educated, rural, and culturally diverse samples. We did not assess formal measurement equivalence across ethnic groups.

### 2.6. Statistical Analysis

Continuous variables were summarized as means and standard deviations and categorical variables as frequencies and percentages. Between-group comparisons by sex and ethnicity used independent-samples *t* tests or one-way ANOVA for continuous variables, as appropriate. Categorical variables were compared using Pearson’s chi-square test when its assumptions were satisfied. Fisher’s exact test was used for sparse 2 × 2 contingency tables, whereas the Fisher–Freeman–Halton exact test was used for larger contingency tables with small expected cell frequencies. Spearman correlations examined associations among sociodemographic, clinical, psychosocial, cognitive, functional, and affective measures.

Logistic regression models explored factors associated with cognitive impairment. Variables significant in univariate analyses or supported by prior literature were entered into multivariable models. Model fit was assessed with the Hosmer–Lemeshow test and explanatory power with Cox and Snell and Nagelkerke pseudo-R^2^. Odds ratios (ORs) with 95% confidence intervals (CIs) are reported. Ethnicity and rural residence were not entered simultaneously because they showed near-complete overlap (97.1% concordance), which would have introduced substantial multicollinearity. Consequently, their independent contributions could not be reliably estimated in this sample.

Canonical correlation analysis (CCA) was conducted as an exploratory, hypothesis-generating analysis to characterize multivariate patterns of covariation between social-health determinants (age, sex, recorded disease burden, medication burden, cigarette and alcohol use, discrimination composite score, and social violence composite score) and neurocognitive outcomes (six RUDAS domains, depressive symptoms, and subjective cognitive complaints). Because the RUDAS total score is an exact linear composite of the six domain scores, it was not entered as an additional independent variable in the canonical solution; instead, its correlation with each canonical variate was calculated as a supplementary indicator of global cognitive performance. This allowed the canonical patterns to be examined in terms of both domain-specific and global cognitive associations without introducing linear redundancy. Given the modest sample size relative to the dimensionality of the analysis, canonical functions were interpreted as exploratory, descriptive patterns rather than as confirmatory evidence of stable latent structures. Functions were evaluated using Wilks’ Λ, standardized canonical coefficients, absolute canonical loadings ≥ 0.30, and redundancy indices.

Statistical results were visualized using scatter plots, correlation heatmaps, reliability plots, and graphical representations of the canonical correlation results, as appropriate to each analysis. For the scatter plots of age and RUDAS scores, a small amount of jitter was applied to discrete RUDAS values to reduce overplotting. Jittering was used exclusively for graphical visualization and did not alter the underlying values used in statistical analyses. Analyses were conducted using IBM SPSS Statistics, version 25.0 (IBM Corp., Armonk, NY, USA), and Python, version 3.14.6 (Python Software Foundation, Wilmington, DE, USA); significance was set at α = 0.05. Effect sizes were reported as Cohen’s *d*, Cramér’s V, or phi (ϕ).

### 2.7. Ethical Considerations

The study followed the Declaration of Helsinki and was approved by the Human Subjects Research Ethics Committee of the Universidad San Francisco de Quito (CEISH-USFQ; 2022-001M/2022-001IN; HUBI: 17495) on 27 September 2022. Written informed consent was obtained from all participants. For illiterate participants, consent was obtained orally in the presence of a witness and documented by fingerprint.

## 3. Results

### 3.1. Sociodemographic Factors by Sex Groups

The sample included 16 males (21.3%) and 59 females (78.7%), with mean age 72.32 years (SD = 6.03). Age did not differ by sex (*p* = 0.614), nor did other sociodemographic variables. Most participants were married (58.7%) or widowed (22.7%) and living with a partner or family member (82.7%). Ethnic composition and health insurance coverage were similar in males and females (all *p* > 0.30; Table 1).

Lifestyle and care-related factors were also similar by sex except for smoking and alcohol use. Physical activity did not differ (*p* = 0.741). Males were more likely to report smoking history (56.3% vs. 15.3%; *p* = 0.002; ϕ = 0.39) and alcohol use (56.3% vs. 15.3%; *p* = 0.002; ϕ = 0.39). Lack of medical attention, discrimination exposure and scores, social violence exposure, and social violence composite score severity did not differ by sex (all *p* > 0.25).

### 3.2. Clinical Status by Sex Groups

Cognitive status distribution did not differ by sex (*p* = 0.486): 77.3% of participants were cognitively normal, 12.0% had MCI, and 10.7% had dementia. Dementia was more frequent in males than females (18.8% vs. 8.5%), but the difference was not significant. RUDAS total scores did not differ by sex (*p* = 0.597). Subjective cognitive decline was more frequent in females (74.6% vs. 50.0%; *p* = 0.072), and females reported more subjective complaints (*p* = 0.087), but neither reached significance. No significant sex differences were observed in memory, visuospatial orientation, praxis, visuoconstruction, or judgment (all *p* > 0.60). Females outperformed males only in the language domain (*p* = 0.038; *d* = 0.59) (Table 2).

### 3.3. Functional, Social Activity, and Mental Health Across Different Sex Groups

Functional impairment on the Pfeffer FAQ, family functionality, and recent stress did not differ by sex (*p* = 1.000, *p* = 0.942, and *p* = 0.100, respectively). Depressive symptoms were higher in females than in males (*p* = 0.006; *d* = 0.79) (Table 2).

### 3.4. Demographic and Living Conditions by Ethnicity

Subgroup analyses by ethnicity included 35 Mestizo, 35 Indigenous, and 5 Other participants. Sex distribution did not differ across ethnic groups (χ^2^ = 0.09, *p* = 0.956). Living arrangements also did not differ significantly (χ^2^ = 10.99, *p* = 0.202; V = 0.27). In contrast, geographic segregation by ethnicity was almost complete (χ^2^ = 62.42, *p* < 0.001; V = 0.91): 97.1% of Indigenous participants lived in rural areas, whereas 97.1% of Mestizo participants lived in urban Cotacachi. Educational access showed the same pattern: 85.7% of Indigenous participants had never attended school, whereas 85.7% of Mestizo participants attended public school. Type of school differed markedly by ethnicity (χ^2^ = 51.35, *p* < 0.001; V = 0.59), and attendance at rural primary school was also associated with ethnicity (χ^2^ = 8.24, *p* = 0.016; V = 0.33). Low educational attainment or illiteracy was highly prevalent across groups and did not differ significantly (χ^2^ = 3.57, *p* = 0.168; V = 0.22), nor did child labor (χ^2^ = 1.40, *p* = 0.498; V = 0.14).

### 3.5. Healthcare Access and Structural Stressors

Use of public healthcare services did not differ across ethnic groups (χ^2^ = 3.83, *p* = 0.148; V = 0.23). Indigenous participants more often reported inability to access healthcare during the previous 12 months (34.3%) than Mestizo (14.3%) and Other (20.0%) participants, although this difference was not significant (χ^2^ = 3.88, *p* = 0.143; V = 0.23). Perceived discrimination and exposure to social violence were reported in all groups without significant ethnic differences (χ^2^ = 1.91, *p* = 0.386; V = 0.16 and χ^2^ = 3.99, *p* = 0.136; V = 0.23, respectively) (Supplementary Table S1).

### 3.6. Clinical Conditions by Ethnicity

Any chronic disease differed significantly by ethnicity (χ^2^ = 8.96, *p* = 0.011; V = 0.35), with the highest prevalence among Mestizo participants (88.6%), followed by Other (80.0%) and Indigenous participants (57.1%). Hypertension also differed significantly across groups (χ^2^ = 10.52, *p* = 0.005; V = 0.37), whereas diabetes mellitus did not (χ^2^ = 3.62, *p* = 0.164; V = 0.22). Polypharmacy (≥3 medications) did not differ by ethnicity (χ^2^ = 3.88, *p* = 0.143; V = 0.23), with frequencies of 34.3% among Mestizo, 14.3% among Indigenous, and 20.0% among Other participants. Medication non-adherence also did not differ significantly across groups (χ^2^ = 0.78, *p* = 0.677; V = 0.10). Functional impairment according to the Pfeffer FAQ was observed in 3/35 (8.6%) Indigenous participants, 1/35 (2.9%) Mestizo participants, and 2/5 (40.0%) participants in the Other group. Given the sparse cell frequencies, the association was evaluated using the Fisher–Freeman–Halton exact test and did not reach statistical significance (*p* = 0.063).

### 3.7. Lifestyle and Health Behaviors by Ethnicity

Smoking history differed by ethnicity (χ^2^ = 6.70, *p* = 0.035; V = 0.30), with the highest prevalence among Mestizo participants (37.1%) and no cases in the Other group. Alcohol use and frequent binge drinking did not differ significantly (χ^2^ = 3.51, *p* = 0.173; V = 0.22 and χ^2^ = 2.80, *p* = 0.247; V = 0.19, respectively). Sedentary lifestyle was also similar across groups (χ^2^ = 4.41, *p* = 0.110; V = 0.24). Recent weight loss differed significantly across groups (χ^2^ = 7.38, *p* = 0.025; V = 0.31), being most frequent among Other (80.0%) and Indigenous participants (48.6%).

### 3.8. Preliminary Psychometric Evaluation of the RUDAS in the Cotacachi Sample

All six RUDAS domains correlated positively with the corrected total RUDAS score (r = 0.54–0.84, all *p* < 0.001), supporting internal coherence between domain-level performance and global cognitive performance. The strongest correlation was observed for Language (r = 0.84), followed by Memory (r = 0.63), Visuoconstruction (r = 0.62), Praxis (r = 0.62), Judgment (r = 0.60), and Orientation (r = 0.54). Inter-domain correlations ranged from −0.06 to 0.50 and were generally small to moderate, with only a negligible negative association, indicating related but non-redundant cognitive domains (Supplementary Figure S1A).

### 3.9. RUDAS Reliability

RUDAS showed moderate internal consistency, with Cronbach’s alpha of 0.663 (standardized α = 0.737) and McDonald’s omega of 0.82 (Supplementary Figure S1B). Spearman correlations provided preliminary evidence consistent with the expected construct-related validity pattern (Supplementary Figure S2). Age was inversely associated with RUDAS scores (ρ = −0.41, *p* < 0.01) and positively associated with less education (ρ = 0.35, *p* < 0.01). Sex was positively associated with cigarette use (ρ = 0.39, *p* < 0.01), alcohol use (ρ = 0.28, *p* < 0.05), and depressive symptoms (ρ = 0.34, *p* < 0.01). Functional impairment was associated with poorer family functioning (ρ = 0.33, *p* < 0.01), higher depressive symptoms (ρ = 0.24, *p* < 0.05), and lower RUDAS scores (ρ = −0.31, *p* < 0.01). Depressive symptoms were inversely associated with RUDAS scores (ρ = −0.40, *p* < 0.01).

Education showed a strong association with RUDAS performance: less education correlated with lower RUDAS scores (ρ = −0.52, *p* < 0.01), whereas years of education correlated positively (ρ = 0.52, *p* < 0.001). The negative association between RUDAS performance and the Family Questionnaire (ρ = −0.22, *p* < 0.05) was consistent with preliminary convergent validity. Conversely, weak or non-significant associations with discrimination, social violence, substance use, and physical activity were consistent with preliminary discriminant validity. Participants were then classified into cognitive status groups using the low-education Andean cutoffs described in the Methods; the characteristics of the resulting cognitive-status groups are presented in Supplementary Table S2. In exploratory analyses stratified by ethnicity, internal-consistency estimates were α = 0.631, standardized α = 0.646, and ω = 0.773 among Indigenous participants, compared with α = 0.555, standardized α = 0.715, and ω = 0.806 among Mestizo participants. These subgroup estimates were considered descriptive given the modest sample sizes and were not interpreted as evidence of formal measurement invariance.

### 3.10. Social Exposure Across Cognitive Status Groups

Discrimination and social violence did not differ significantly across cognitive status groups (*p* = 0.417 and *p* = 0.159, respectively) (Supplementary Table S2).

### 3.11. Mental Health and Perception Across Cognitive Status Groups

Subjective cognitive decline was common across groups and did not differ by cognitive status (*p* = 0.428). Depressive symptom severity also did not differ significantly (*p* = 0.118), although mild symptoms were more frequent in MCI and dementia. Recent stress levels were similar across cognitive groups (*p* = 0.977) (Supplementary Table S2).

### 3.12. Cognitive Status by Ethnicity

Cognitive status was significantly associated with ethnicity (χ^2^(4) = 10.80, *p* = 0.029; Cramér’s V = 0.27). Given the small, expected frequencies in several cells, the association was additionally evaluated using the Fisher–Freeman–Halton exact test (*p* = 0.010). Indigenous participants accounted for a larger proportion of MCI and dementia cases, whereas cognitively normal status was more frequent among Mestizo participants (Figure 1; Supplementary Table S2).

In multivariable logistic regression, age and depressive symptoms remained associated with cognitive impairment after adjustment. Each additional year of age increased the odds by 17% (OR = 1.17; 95% CI: 1.04–1.31; *p* = 0.007), and each one-point increase in Yesavage score increased the odds by 33% (OR = 1.33; 95% CI: 1.05–1.67; *p* = 0.017). Sex was not significantly associated with cognitive impairment after adjustment (OR = 0.31; 95% CI: 0.07–1.44; *p* = 0.134) (see Supplementary Table S3). Exploratory adjusted analyses showed no statistically significant association between recorded disease burden and cognitive impairment (OR = 0.52, 95% CI: 0.25–1.07; *p* = 0.075). Medication count, hypertension, diabetes, arrhythmia, cardiometabolic conditions, neurological history, discrimination, violence exposure, and other comorbidities were also not significantly associated.

The final model was statistically significant (χ^2^(3) = 17.21, *p* < 0.001), showed adequate calibration (Hosmer–Lemeshow χ^2^(8) = 9.12, *p* = 0.333), and explained approximately 31% of the variance in cognitive impairment (Nagelkerke R^2^ = 0.312). Exploratory models including either rural residence or ethnicity separately yielded borderline and similar estimates; however, because of their near-complete overlap, these models should not be interpreted as estimating independent effects.

### 3.13. Exploratory Canonical Associations Between Social-Health Determinants and Neurocognitive Outcomes

The exploratory CCA yielded two statistically significant canonical functions (Figure 2). Given the modest sample size relative to the dimensionality of the analysis, these functions were interpreted as descriptive, hypothesis-generating patterns of covariation rather than as confirmatory evidence of stable underlying structures.

### 3.14. Function 1: Psychosocial–Affective Pattern

The first function was statistically significant (Rc = 0.712; Wilks’ Λ = 0.175; *p* < 0.001) but showed modest redundancy, explaining 4.9% of the variance in Set 2. The principal loadings in Set 1 were sex (0.58), discrimination composite score (0.44), and medication burden (0.44), whereas depressive symptoms (0.65) and RUDAS Item 6 (Language; 0.51) showed the highest loadings in Set 2. The supplementary correlation of the corrected RUDAS total score with the corresponding canonical variate was weak (r = 0.15), suggesting that this pattern was more closely related to specific cognitive and affective features than to global cognitive performance. Given the exploratory nature of the analysis, this function was interpreted as a psychosocial–affective pattern rather than as a stable latent dimension.

### 3.15. Function 2: Aging and Structural Exposure Pattern

The second function was also statistically significant (Rc = 0.646; Wilks’ Λ = 0.355; *p* = 0.038) and explained 10.2% of the variance in Set 2. The strongest loadings in Set 1 were age (0.62), social violence composite score (0.54), recorded disease burden (−0.40), alcohol use (0.37), and cigarette use (−0.33). In Set 2, the principal contributors were RUDAS Item 3 (Visuoconstruction; −0.95), RUDAS Item 2 (Praxis; −0.61), depressive symptoms (0.48), RUDAS Item 1 (Orientation; −0.42), and RUDAS Item 6 (Language; −0.36). The supplementary correlation of the corrected RUDAS total score with the corresponding canonical variate was substantial (r = −0.65), indicating that this pattern was associated with both global cognitive performance and specific cognitive domains, particularly visuoconstruction and praxis. Given the exploratory nature of the analysis, this function was interpreted as an aging and structural-exposure pattern rather than as a stable latent dimension.

## 4. Discussion

This study examined cognitive aging within a rural Andean setting in which Indigenous identity, territorial disadvantage, limited early-life educational opportunities, and restricted healthcare access strongly overlap. The contribution of the study lies primarily in characterizing how these structural and life-course conditions converge within the Cotacachi population. Three findings were particularly relevant: cognitive impairment was more frequently identified among Indigenous participants; educational and territorial disadvantage were strongly patterned by ethnicity; and age and depressive symptoms remained the principal adjusted correlates of cognitive impairment (Figure 3).

### 4.1. Rurality and Territorial Structuring of Cognitive Health Disparities

The near-complete overlap between ethnicity and place of residence indicates that cognitive disparities in this cohort are territorially structured. Almost all Indigenous participants lived in rural areas, whereas almost all Mestizo participants lived in the urban zone. Rurality in this context reflects more than geography: it marks historical exclusion from education, healthcare, and infrastructure. The fact that 85.7% of Indigenous participants had never attended school, compared with 85.7% of Mestizo participants who attended public school, suggests that ethnicity in this setting is best interpreted as a proxy for long-term exposure to territorially patterned disadvantage rather than intrinsic vulnerability.

### 4.2. Early-Life Education as a Core Determinant of Cognitive Performance

Educational deprivation emerged as a key determinant of cognitive performance. Indigenous participants were disproportionately exposed to limited or absent formal schooling, and lower educational attainment was strongly associated with lower RUDAS scores. This finding is consistent with evidence showing that rural childhood residence and limited schooling are associated with poorer memory, language, and executive function later in life [27,28,29,30]. Previous studies in rural Ecuador have similarly shown that cognitive aging is embedded within a combination of social, clinical, and vascular conditions [9,16]. Evidence from Peru and rural–urban comparisons in Mexico further indicates that sociodemographic context, lifestyle, and place of residence contribute to differences in cognitive performance and cognitive aging [29,30]. Cotacachi represents a particularly marked expression of these contextual inequalities because Indigenous identity, rural residence, and severe educational disadvantage strongly overlap within the same population.

This pattern is also consistent with cognitive reserve theory, according to which limited education may reduce resilience to age-related and pathological cognitive decline [31,32,33,34]. Accordingly, the higher frequency of participants classified as having MCI or dementia among Indigenous participants should be interpreted within this broader context of cumulative educational and territorial disadvantage rather than as evidence of an intrinsic or independent effect of ethnicity.

Although the RUDAS was selected because it is less influenced by formal education than several conventional cognitive screening instruments [21,22], it should not be considered education-independent. Measurement equivalence between Indigenous and Mestizo participants was not formally evaluated in this study. Therefore, lower RUDAS performance among Indigenous participants should not, by itself, be interpreted as evidence of a higher underlying prevalence of neurodegenerative disease, particularly given the marked differences in early-life educational exposure between groups. Cognitive classification incorporated functional assessment and DSM-5 criteria in addition to education-adjusted RUDAS thresholds, reducing reliance on cognitive screening scores alone. The internal consistency estimates observed in the present sample were broadly comparable to those reported in previous Latin American RUDAS studies and should be interpreted within the broader evidence supporting the use of culturally appropriate cognitive screening tools in low-educated and illiterate populations [18,21,22]. Nevertheless, these comparisons do not establish psychometric validity in the present population, and the current findings should be regarded as preliminary, given the modest sample size and the absence of formal measurement invariance testing. The very limited schooling experienced by many older Indigenous participants reflects educational conditions affecting earlier cohorts and should not be assumed to represent current educational opportunities in Cotacachi. Nevertheless, persistent rural and Indigenous inequalities in access to educational and social resources remain relevant when considering future trajectories of cognitive aging. Consistent with this cautious interpretation, functional impairment was numerically more frequent among Indigenous than Mestizo participants, but the difference was not statistically significant, indicating that between-group differences in cognitive screening performance were not paralleled by a clear difference in functional impairment.

### 4.3. Accumulation of Social Disadvantage and Cognitive Vulnerability

Indigenous participants also showed greater barriers to healthcare and greater exposure to social violence, within a broader profile of discrimination and cumulative social disadvantage. Although many of these factors were not independently significant in adjusted cross-sectional models, their direction and effect sizes suggest a broader pattern compatible with the “social exposome,” in which repeated social adversity may contribute to unfavorable cognitive aging trajectories. These disparities are better understood as the downstream expression of historical and structural inequities operating across the life course.

### 4.4. Health Conditions as Potential Mediators

Differences in recorded chronic disease burden, polypharmacy, smoking history, and recent weight loss provide plausible pathways linking social disadvantage to cognitive vulnerability. Similar patterns of social and contextual disadvantage have been described across Latin American populations [35,36,37,38,39,40,41]. Studies in rural and Indigenous populations also document multimorbidity, cardiometabolic risk, frailty, and underdiagnosis [42,43,44,45,46,47].

However, the lower recorded frequency of selected clinical conditions among Indigenous participants should not be interpreted as evidence of a lower underlying disease burden. Because these variables reflect documented or reported medical history rather than systematic ascertainment of previously undiagnosed disease, restricted healthcare access may reduce opportunities for detection, treatment, and longitudinal follow-up. This is particularly relevant for modifiable vascular and metabolic conditions such as hypertension, diabetes, and dyslipidemia, which may remain clinically silent for prolonged periods. Similarly, lower polypharmacy may reflect reduced access to diagnosis and treatment rather than better health. Accordingly, disease burden in this population should be interpreted as documented disease burden rather than necessarily representing the true underlying prevalence of these conditions.

More broadly, the vascular and lifestyle context of rural Ecuador should be interpreted within a changing social and epidemiological environment. Recent evidence from transitional rural Ecuadorian communities has documented a substantial burden of cardiometabolic disease and associated modifiable risk factors, including hypertension, diabetes, adiposity, smoking, and alcohol exposure [45]. These findings suggest that vascular and lifestyle risks remain relevant in rural populations undergoing social and lifestyle transitions and may continue to shape future trajectories of cognitive aging.

### 4.5. Ethnicity as a Marker of Structural Inequity

Although cognitive impairment was more frequent among Indigenous participants, the near-complete overlap between Indigenous identity and rural residence prevented their independent contributions from being reliably disentangled in adjusted analyses. This pattern is consistent with regional evidence suggesting that dementia risk in Latin America is strongly shaped by social and contextual determinants rather than by genetic ancestry alone [12,35]. In the present cohort, ethnicity should therefore be interpreted primarily as a marker of territorially patterned social disadvantage rather than as an independent or intrinsic determinant of cognitive vulnerability.

Age remained the principal adjusted correlate of cognitive impairment, while depressive symptoms represented the other factor retained in adjusted analyses. Depression is a well-established correlate of incident dementia and accelerated cognitive decline in socially vulnerable populations [48,49] and may represent a prodromal manifestation, a contributing factor, or a marker of reduced cognitive reserve. The exploratory canonical correlation analysis identified two complementary patterns of multivariate covariation. The first, characterized by sex, discrimination, medication burden, depressive symptoms, and language performance, showed only a weak supplementary association with the RUDAS total score, suggesting a pattern more closely related to specific cognitive and affective features than to global cognition. In contrast, the second pattern, characterized by older age and greater exposure to social violence together with poorer visuoconstruction, praxis, orientation, and language performance, showed a substantial supplementary association with the RUDAS total score, suggesting associations with both global and domain-specific cognitive variation. Given the modest sample size, the dimensionality of the analysis, and the relatively low-to-moderate redundancy of the two functions, these patterns should be considered exploratory and hypothesis-generating rather than stable latent structures. Within this framework, the findings are consistent with a possible interplay between biological aging, affective symptoms, and cumulative social disadvantage.

Recent nationally representative evidence from Ecuador provides additional context for this interpretation. Dementia prevalence was 11.6% overall, but was substantially higher among Indigenous than non-Indigenous older adults (21.9% vs. 11.1%) and among rural than urban residents (14.3% vs. 11.0%) [50]. In that study, rural residence and associated contextual characteristics accounted for a substantial proportion of the Indigenous–non-Indigenous difference. Although the present Cotacachi sample is not nationally representative, the close overlap between Indigenous identity and rural residence observed here is consistent with this broader Ecuadorian pattern.

## 5. Limitations

The modest sample size limited statistical power, particularly for low-frequency clinical conditions and subgroup comparisons, including analyses involving the small and heterogeneous Other subgroup. Accordingly, some between-group findings should be considered exploratory and require replication in larger and more heterogeneous samples. The sample size was also limited relative to the dimensionality of the canonical correlation analysis, which may affect the stability of the canonical coefficients, loadings, and redundancy estimates. These multivariate patterns should therefore be regarded as hypothesis-generating and potentially sample-dependent pending confirmation in larger datasets. The cross-sectional design precluded causal inference and temporal ordering, particularly regarding the relationship between depressive symptoms and cognitive impairment. In addition, the near-complete overlap between Indigenous identity and rural residence prevented their independent contributions from being reliably disentangled. The relative contextual homogeneity of the study setting may also have attenuated associations with some social and environmental factors.

Finally, the psychometric evaluation of the RUDAS in the present sample was preliminary. Measurement equivalence between Indigenous and Mestizo participants was not formally assessed; therefore, between-group differences in cognitive screening performance should be interpreted cautiously, particularly given the substantial differences in educational exposure between groups.

## 6. Future Research Directions

Future studies should include larger and more heterogeneous rural, peri-urban, and urban samples, with longitudinal follow-up to clarify the temporal relationships among education, depression, chronic disease, and cognitive decline. Extended neurocognitive batteries, biomarkers, vascular measures, and social exposome indicators would clarify mechanisms beyond screening. At the policy level, Ecuador would benefit from a coordinated dementia strategy with culturally adapted tests, standardized training, and equitable implementation in Indigenous and underserved territories.

## 7. Conclusions

In this population-based sample of older adults from Cotacachi, cognitive impairment was more frequently identified among Indigenous participants, but Indigenous identity, rural residence, and educational disadvantage were so closely intertwined that their independent contributions could not be reliably disentangled. Older age and depressive symptoms emerged as the principal adjusted correlates of cognitive impairment, whereas recorded vascular and clinical conditions were not independently associated. These findings support interpreting observed cognitive disparities within a broader context of cumulative educational, territorial, and social disadvantage rather than as an intrinsic effect of ethnicity. The psychometric findings for the RUDAS and the canonical correlation patterns should be considered preliminary and exploratory, respectively, and require confirmation in larger and more heterogeneous populations.

## Figures and Tables

**Figure 1:**
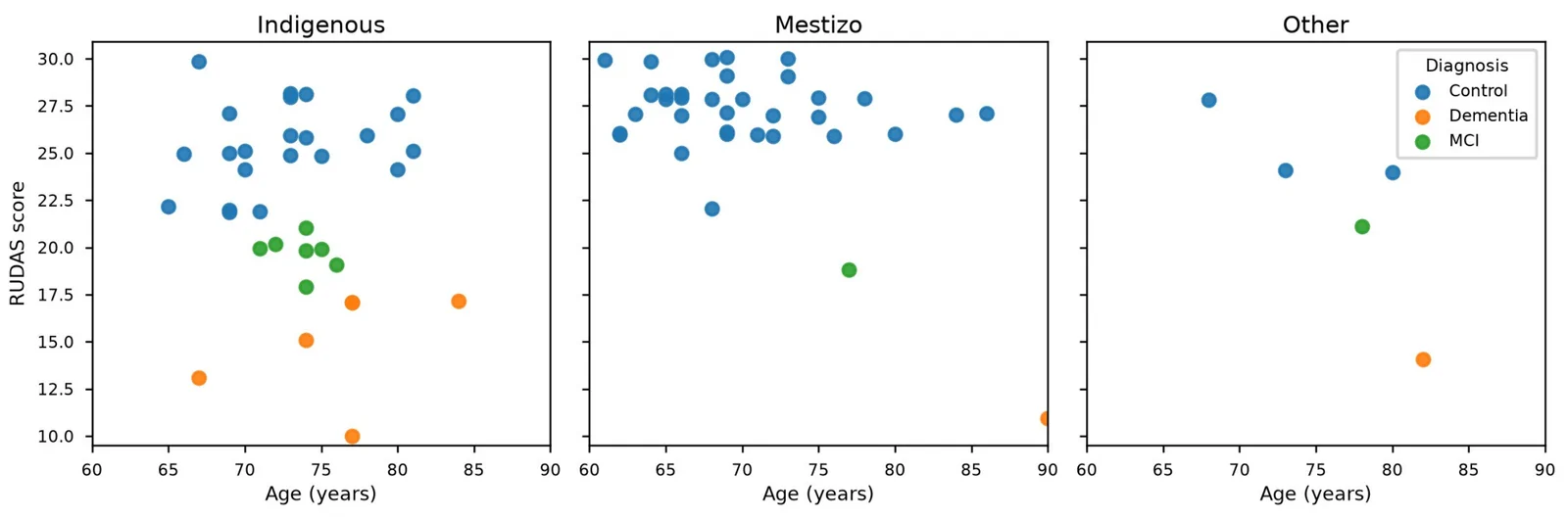
Association between age and RUDAS score stratified by participant group. Scatter plots are shown separately for Indigenous, Mestizo, and Other participants, with points differentiated according to cognitive-status group.

**Figure 2:**
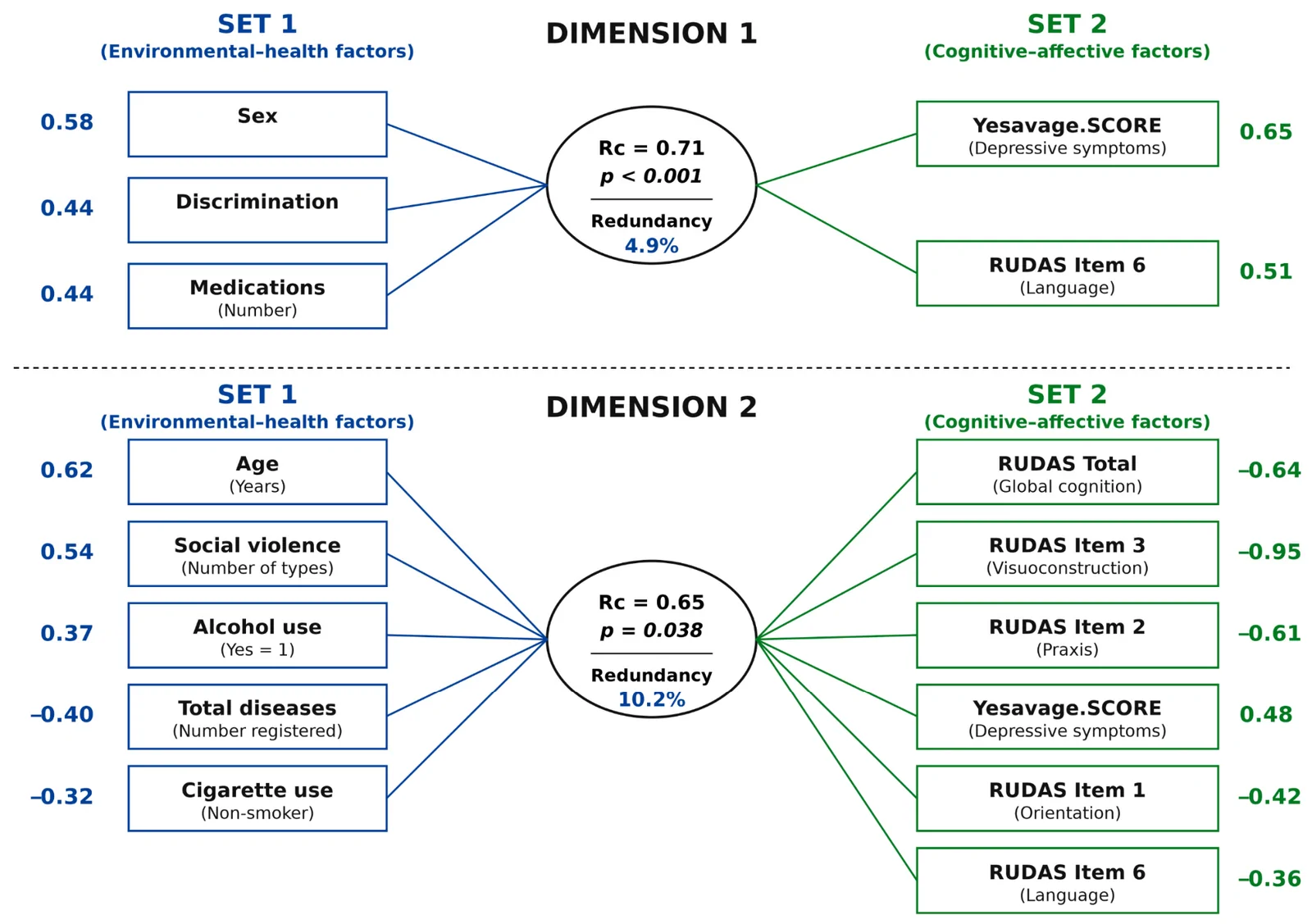
Exploratory canonical correlation analysis between social–health determinants and neurocognitive outcomes. Dimension 1 (Psychosocial–Affective Pattern) links sex, discrimination, and medication burden with depressive symptoms and language performance. Dimension 2 (Aging and Structural Exposure Pattern) links age, social violence, alcohol use, recorded disease burden, and cigarette use with cognitive performance, particularly visuoconstruction, praxis, orientation, and language. The corrected RUDAS total score is displayed as a supplementary indicator of global cognitive performance and was not entered as an additional variable in the canonical solution because it is an exact linear composite of the six RUDAS domains. Rc denotes the canonical correlation coefficient; redundancy represents the proportion of variance in Set 2 accounted for by each canonical function. Only variables with absolute canonical loadings ≥ 0.30 are displayed. Blue represents Set 1 (environmental–health factors), and green represents Set 2 (cognitive–affective factors).

**Figure 3:**
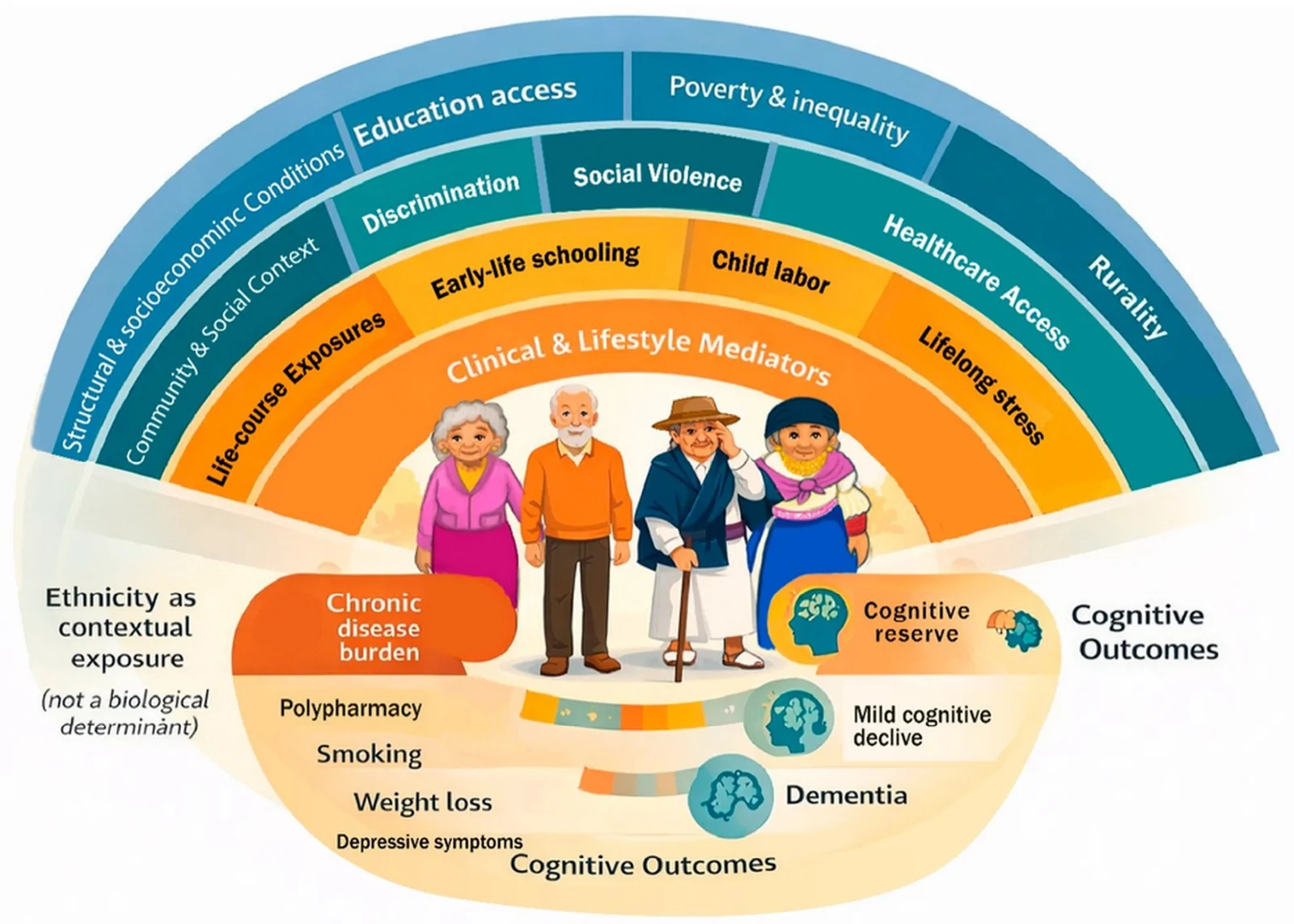
Structural Determinants and Cumulative Vulnerability Shaping Cognitive Aging. The diagram illustrates how structural determinants (outer layers) condition life experiences and clinical mediators (middle layers), shaping cognitive reserve and the risk of decline (center). Ethnicity is emphasized as a marker of contextual exposure and accumulated structural vulnerability rather than as an intrinsic biological determinant, consistent with the finding that age and depressive symptom burden were the principal adjusted correlates of cognitive status. Blue and teal indicate structural and community-level determinants, whereas orange and yellow indicate life-course exposures and clinical/lifestyle mediators. The multicolored central band is decorative and does not represent additional variables or categories. Visual design was developed with the assistance of ChatGPT (GPT-5.6 Sol, OpenAI, San Francisco, CA, USA) and refined using Adobe Illustrator 2023 and Adobe Photoshop 2023.

**Table 1:** Sociodemographic, lifestyle, social exposure, and clinical characteristics of the study sample by sex.

Variables		Total (N = 75)	Male (*n* = 16)	Female (*n* = 59)	F/χ^2^	t	*p*	Effect Size
Sociodemographic	Age (years), M (SD)	72.32 (6.03)	73.00 (6.50)	72.14 (5.94)	0.31	0.51	0.614	*d* = 0.14
	Handedness (right), *n* (%)	71 (94.7)	16 (100)	55 (93.2)	—	—	0.572	ϕ = 0.12
Marital status, *n* (%)	Married	44 (58.7)	12 (75.0)	32 (54.2)	5.30	—	0.381	V = 0.27
	Widowed	17 (22.7)	3 (18.8)	14 (23.7)				
	Other (div./sep./cohab.)	14 (18.6)	1 (6.2)	13 (22.1)				
Living arrangement, *n* (%)	Alone	13 (17.3)	2 (12.5)	11 (18.6)	5.95	—	0.203	V = 0.28
	With partner/family	62 (82.7)	14 (87.5)	48 (81.4)				
Ethnicity, *n* (%)	Indigenous	35 (46.7)	7 (43.8)	28 (47.5)	4.86	—	0.302	V = 0.26
	Mestizo	35 (46.7)	8 (50.0)	27 (45.8)				
Ethnic self-identification, *n* (%)	Able to answer	48 (64.0)	9 (56.3)	39 (66.1)	1.40	—	0.498	V = 0.14
Health insurance, *n* (%)	Public (MSP)	40 (53.3)	9 (56.3)	31 (52.5)	0.33	—	0.847	V = 0.07
Lifestyle & care	Physical activity, M (SD)	2.69 (0.95)	2.75 (0.93)	2.66 (0.96)	0.28	0.33	0.741	*d* = 0.09
	Smoking history (yes), *n* (%)	18 (24.0)	9 (56.3)	9 (15.3)	—	—	0.002 **	ϕ = 0.39
	Alcohol history (yes), *n* (%)	18 (24.0)	9 (56.3)	9 (15.3)	—	—	0.002 **	ϕ = 0.39
	Medical attention (no), *n* (%)	57 (76.0)	13 (81.3)	44 (74.6)	—	—	0.747	ϕ = 0.06
Social exposure	Discrimination (yes), *n* (%)	34 (45.3)	7 (43.8)	27 (45.8)	0.02	—	0.886	ϕ = 0.02
	Discrimination score, M (SD)	1.24 (1.57)	1.13 (1.50)	1.27 (1.61)	0.09	−0.33	0.744	*d* = 0.09
	Social violence (yes), *n* (%)	12 (16.0)	4 (25.0)	8 (13.6)	—	—	0.271	ϕ = 0.13
	Violence score, M (SD)	0.39 (1.05)	0.50 (0.89)	0.36 (1.09)	0.33	0.48	0.630	*d* = 0.13
Cognitive status, *n* (%)	Control	58 (77.3)	11 (68.8)	47 (79.7)	1.44	—	0.486	V = 0.14
	MCI	9 (12)	2 (12.5)	7 (11.9)				
	Dementia	8 (10.7)	3 (18.8)	5 (8.5)				

Note. Values are presented as mean (SD) or *n* (%). Group comparisons used independent-samples *t* tests and χ^2^ or Fisher’s exact tests, as appropriate. Effect sizes are reported as Cohen’s *d*, phi (ϕ), or Cramér’s *V*. The Other ethnic category (*n* = 5) is not displayed separately because of its small size; ethnicity statistics were calculated using the original self-identification categories. MCI = mild cognitive impairment; Div. = divorced; Sep. = separated; Cohab. = cohabiting. ** *p* < 0.01.

**Table 2:** Cognitive performance, functional status, psychosocial factors, and mental health measures by sex.

Variable		Total (N = 75)	Male (*n* = 16)	Female (*n* = 59)	F/χ^2^	t	*p*	Effect Size
Global cognitive measures	RUDAS total score, M (SD)	24.43 (4.66)	23.88 (6.41)	24.58 (4.13)	7.64	−0.53	0.597	*d* = 0.13
	SCD (subjective complaints), *n* (%)	52 (69.3)	8 (50.0)	44 (74.6)	—	—	0.072	ϕ = 0.22
	Total subjective complaints, M (SD)	0.99 (0.95)	0.63 (0.81)	1.08 (0.97)	0.02	−1.74	0.087	*d* = 0.48
RUDAS cognitive domains	Visuospatial orientation, M (SD)	4.73 (0.60)	4.69 (0.60)	4.75 (0.60)	0.21	−0.34	0.733	*d* = 0.10
	Praxis, M (SD)	1.56 (0.58)	1.50 (0.63)	1.58 (0.56)	0.53	−0.47	0.641	*d* = 0.14
	Visuoconstruction, M (SD)	1.56 (1.17)	1.69 (1.40)	1.53 (1.10)	6.36	0.49	0.625	*d* = 0.14
	Judgment, M (SD)	2.91 (1.39)	2.88 (1.41)	2.92 (1.39)	0.12	−0.10	0.919	*d* = 0.03
	Memory, M (SD)	6.37 (1.91)	6.50 (1.86)	6.34 (1.94)	0.07	0.30	0.767	*d* = 0.08
	Language, M (SD)	7.29 (1.46)	6.63 (2.39)	7.47 (1.04)	20.46	−2.11	0.038 *	*d* = 0.59
Functional & psychosocial measures	Pfeffer FAQ (impaired), *n* (%)	6 (8.0)	1 (6.2)	5 (8.5)	0.08	—	1.000 a	ϕ = 0.03
	Family functionality (high), *n* (%)	24 (32.0)	5 (31.3)	19 (32.2)	0.005	—	0.942	ϕ = 0.01
Recent stress level, *n* (%)	Low/no stress	33 (44.0)	11 (68.7)	22 (37.3)	7.78	—	0.100	V = 0.32
	Moderate/high stress	42 (56.0)	5 (31.3)	37 (62.7)				
Mental health	Yesavage score (GDS), M (SD)	4.63 (2.86)	2.94 (1.81)	5.12 (2.95)	4.88	−2.81	0.006 **	*d* = 0.79

Note. Values are presented as mean (SD) or *n* (%). Group comparisons were performed using independent-samples *t* tests or χ^2^ tests, as appropriate. Effect sizes are reported as Cohen’s *d*, phi (ϕ), or Cramér’s *V*. RUDAS = Rowland Universal Dementia Assessment Scale; SCD = subjective cognitive decline; FAQ = Functional Activities Questionnaire; GDS = Geriatric Depression Scale. a: Fisher’s exact test. * *p* < 0.05; ** *p* < 0.01.

## Data Availability

The data supporting the findings of this study are available within the article. Additional data may be made available from the corresponding author upon reasonable request, subject to ethical and institutional restrictions. During the preparation of this work, generative AI and AI-assisted technologies were used to support literature searches, data table structuring, manuscript organization refinement, coding, and the visual design and high-resolution export of Figure 3. Additionally, these tools were employed to improve grammatical accuracy and assist with English translation. Following this process, all content, data visualizations, and references were critically reviewed and validated by the authors, who maintain full responsibility for the original ideas, the accuracy of the data, and the scientific integrity of the final manuscript.

## Supplementary Materials

The following supporting information can be downloaded at: https://www.mdpi.com/article/10.3390/brainsci16101002/s1, Figure S1: Psychometric properties of the RUDAS: (A) inter-domain and domain–total correlations; (B) internal consistency and reliability estimates; Figure S2: Heatmap of Spearman correlation coefficients among sociodemographic, clinical, psychosocial, and cognitive variables; Table S1: Early Life, Social, Clinical, and Lifestyle Determinants by Ethnicity; Table S2: Social exposure, mental health, and perceived stress across cognitive status groups; Table S3: Multivariable Binary Logistic Regression Analysis of Factors Associated with Cognitive Impairment.
