# Supplementary material for: Social Determinants of Health and Neurocognitive Aging in Indigenous and Non-Indigenous Older Adults in Rural Andean Ecuador: A Population-Based Study in Cotacachi
Source: Brain Sci. 2026 Sep 22;16(10):1002. doi: 10.3390/brainsci16101002 (PMC13702574; doi:10.3390/brainsci16101002)
Supplement: Supplementary file 1 [file brainsci-16-01002-s001.zip › brainsci-4516356-supplementary.pdf]

Supplementary Table S1. Early Life, Social, Clinical, and Lifestyle Determinants by Ethnicity.

	Variable	Category	Mestizo n (%)	Indigenous n (%)	Other / White n (%)	χ²	p	Effect size
**Demographic & Living Conditions**	Sex	Male	8 (22.9)	7 (20.0)	1 (20.0)	0.09	0.956	0.03
		Female	27 (77.1)	28 (80.0)	4 (80.0)			
	Living arrangement	Alone	5 (14.3)	7 (20.0)	1 (20.0)	10.99	0.202	0.27
		With partner	14 (40.0)	20 (57.1)	2 (40.0)			
		Partner & children	8 (22.9)	1 (2.9)	0 (0.0)			
		With children	5 (14.3)	6 (17.1)	2 (40.0)			
		Other	3 (8.6)	1 (2.9)	0 (0.0)			
	Current Residence	Rural	1 (2.9)	34 (97.1)	2 (40.0)	62.42	**<0.001****	0.91
		Urban	34 (97.1)	1 (2.9)	3 (60.00)			
**Early Life & Educational Determinants**	Type of school attended	Public	30 (85.7)	5 (14.3)	4 (80.0)	51.35	**<0.001****	0.59
		Private	1 (2.9)	0 (0.0)	0 (0.0)			
		Mix	3 (8.6)	0 (0.0)	0 (0.0)			
		None	1 (2.9)	30 (85.7)	1 (20.0)			
	Rural primary school	Yes	12 (34.3)	4 (11.4)	3 (60.0)	8.24	**0.016***	0.33
	Low education/illiteracy	Yes	32 (91.4)	35 (100.0)	5 (100.0)	3.57	0.168	0.22
	Child labor	Yes	33 (94.3)	31 (88.6)	4 (80.0)	1.40	0.498	0.14
**Healthcare Access & Structural Factors**	Public healthcare (MSP)	Yes	17 (48.6)	22 (62.9)	1 (20.0)	3.83	0.14	0.23
	Unable to access healthcare (12 mo)	Yes	5 (14.3)	12 (34.3)	1 (20.0)	3.88	0.143	0.42
	Perceived discrimination	Yes	18 (51.4)	15 (42.9)	1 (20.0)	1.91	0.386	0.16
	Social violence exposure	Yes	3 (8.6)	7 (20.0)	2 (40.0)	3.99	0.136	0.23
**Clinical Conditions**	Any chronic disease	Yes	31 (88.6)	20 (57.1)	4 (80.0)	8.96	**0.011***	0.35
	Hypertension (HTN)	Yes	19 (54.3)	6 (17.1)	2 (40.0)	10.52	**0.005****	0.37
	Diabetes mellitus	Yes	9 (25.7)	3 (8.6)	1 (20.0)	3.62	0.16	0.22
	Polypharmacy (≥3 drugs)	Yes	12 (34.3)	5 (14.3)	1 (20.0)	3.88	0.143	0.23
	Medication non-adherence	Yes	11 (31.4)	8 (22.9)	1 (20.0)	0.78	0.67	0.10
**Lifestyle & Health Behaviors**	Smoking (ever)	Yes	13 (37.1)	5 (14.3)	0 (0.0)	6.70	**0.035***	0.30
	Alcohol consumption (ever)	Yes	21 (60.0)	28 (80.0)	3 (60.0)	3.51	0.173	0.22
	Frequent binge drinking	Yes	6 (17.1)	12 (34.3)	1 (20.0)	2.80	2.47	0.19
	Sedentary lifestyle	Yes	8 (22.9)	3 (8.6)	2 (40.0)	4.41	0.11	0.24
	Recent weight loss	Yes	9 (25.7)	17 (48.6)	4 (80.0)	7.38	**0.025***	0.31

Note. Values are presented as n (%) within each ethnic group. Ethnicity was categorized as Mestizo (n = 35), Indigenous (n = 35), and Other/White (n = 5). The Other/White group includes participants who identify as White or as other non-Indigenous ethnicities. Pearson's chi-square (X^2) tests were used to assess differences across ethnic groups. Effect sizes were estimated using Cramér's V and interpreted as negligible (<0.10), small (0.10–0.29), moderate (0.30–0.49), or large (0.50). Current residence (Rural vs. Urban) revealed a near-total geographic segregation by ethnicity (Mestizo = 97.1% Urban; Indigenous = 97.1% Rural), representing a primary structural determinant in this sample. Due to small cell counts in some comparisons, results should be interpreted as exploratory. Percentages may not sum to 100% due to rounding. *$p < 0.05$; **$p < 0.01$.

Supplementary Table S2. Social exposure, mental health, and perceived stress across cognitive status groups.

Factor		Control (n = 56)	MCI (n = 11)	Dementia (n = 8)	χ^2	p	Effect size
**Social exposure**	Exposed to discrimination (yes), n (%)	26 (46.4)	6 (54.5)	2 (25.0)	1.74	.419	0.15
	Exposed to social violence (yes), n (%)	7 (12.5)	2 (18.2)	3 (37.5)	3.30	.192	0.21
**Mental health & perception**	Subjective cognitive decline (yes), n (%)	40 (71.4)	8 (72.7)	4 (50.0)	1.58	.453	0.15
**Depressive symptoms (Yesavage), n (%)**	Normal (0–5)	40 (71.4)	4 (36.4)	4 (50.0)	6.99	.136	0.22
	Mild depression (6–9)	15 (26.8)	6 (54.5)	4 (50.0)			
	Severe depression (≥10)	1 (1.8)	1 (9.1)	0 (0.0)			
**Recent stress level, n (%)**	Low / no stress	23 (41.1)	6 (54.5)	4 (50.0)	2.98	.935	0.14
	Moderate / high stress	32 (57.1)	5 (45.5)	4 (50.0)			
**Cognitive status by Ethnicity**	Indigenous	21 (37.5)	8 (72.7)	6 (75.0)	9.87	**.043***	0.26[a]
	Mestizo	32 (57.1)	2 (18.2)	1 (12.5)			
	Others	3 (5.4)	1 (9.1)	1 (12.5)			

Note. Values are presented as n (%). Group differences across cognitive status were assessed using χ^2 tests. Effect sizes are reported as Cramér's *V*. MCI = mild cognitive impairment. *$p < 0.05$; **$p < 0.01$. a: Because of small, expected frequencies, the Fisher–Freeman–Halton exact test was also calculated ($p = .020$).

Supplementary Table S3. Multivariable Binary Logistic Regression Analysis of Factors Associated with Cognitive Impairment.

Variable	β (SE)	Adjusted OR	95% CI	p-value
**Age (years)**	0.156 (0.056)	1.17	1.05 – 1.30	**0.005****
**Sex (female vs male)**	-0.883 (0.762)	0.41	0.09 – 1.84	0.247
**Yesavage score**	0.267 (0.114)	1.31	1.04 – 1.63	**0.019***

Note. OR = odds ratio adjusted for all variables included in the model. CI = confidence interval. Cognitive impairment was coded as 1 (impaired) vs 0 (control). *$p < 0.05$; **$p < 0.01$.

A

B

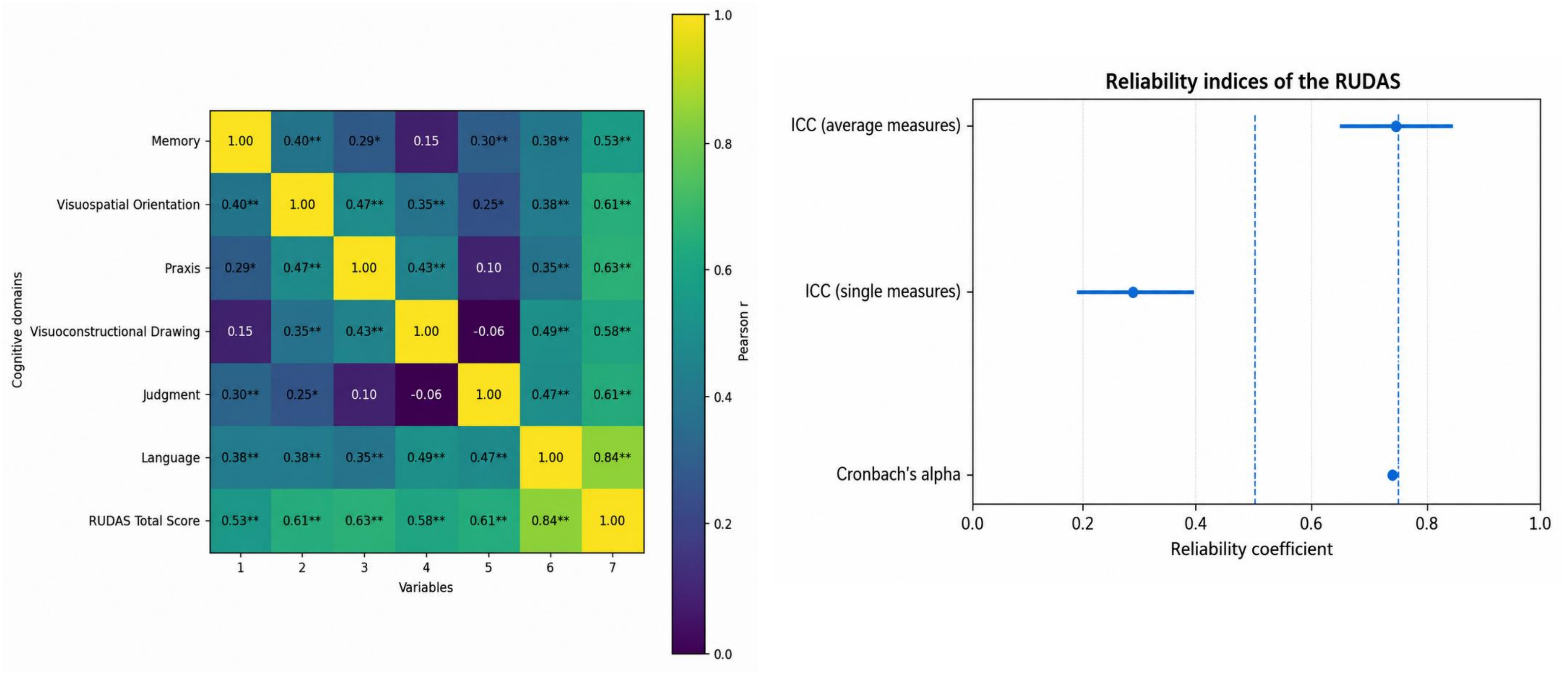

Supplementary Figure S1. Psychometric properties of the RUDAS. **A**, shows a heatmap of Pearson correlations among RUDAS cognitive domains and the total score. Values represent Pearson correlation coefficients (r), with asterisks indicating statistical significance ($p < 0.05$; $p < 0.01$). Cognitive domains are listed on the y-axis, and variables are numbered on the x-axis according to the following order: (1) Memory, (2) Visuospatial Orientation, (3) Praxis, (4) Visuoconstructional Drawing, (5) Judgment, (6) Language, and (7) RUDAS Total Score. The heatmap illustrates moderate to strong positive item–total correlations across all domains, supporting adequate item discrimination and internal coherence of the scale. **B**, presents the reliability indices for the RUDAS, including Cronbach's alpha, McDonald's omega, and intraclass correlation coefficients (ICC) for single and

average measures. Points represent point estimates, and horizontal lines indicate 95% confidence intervals derived from bootstrap resampling (alpha and omega) and mixed-effects modeling (ICC). Dashed vertical lines denote conventional reliability thresholds (0.70 and 0.80), facilitating interpretation of internal consistency and agreement levels. The figure shows moderate internal consistency (Cronbach's alpha), and acceptable-to-good reliability for aggregated RUDAS total scores (ICC average), whereas single-measure reliability was lower. Statistical significance for ICC estimates was set at $p < 0.05$; $p < 0.01$.

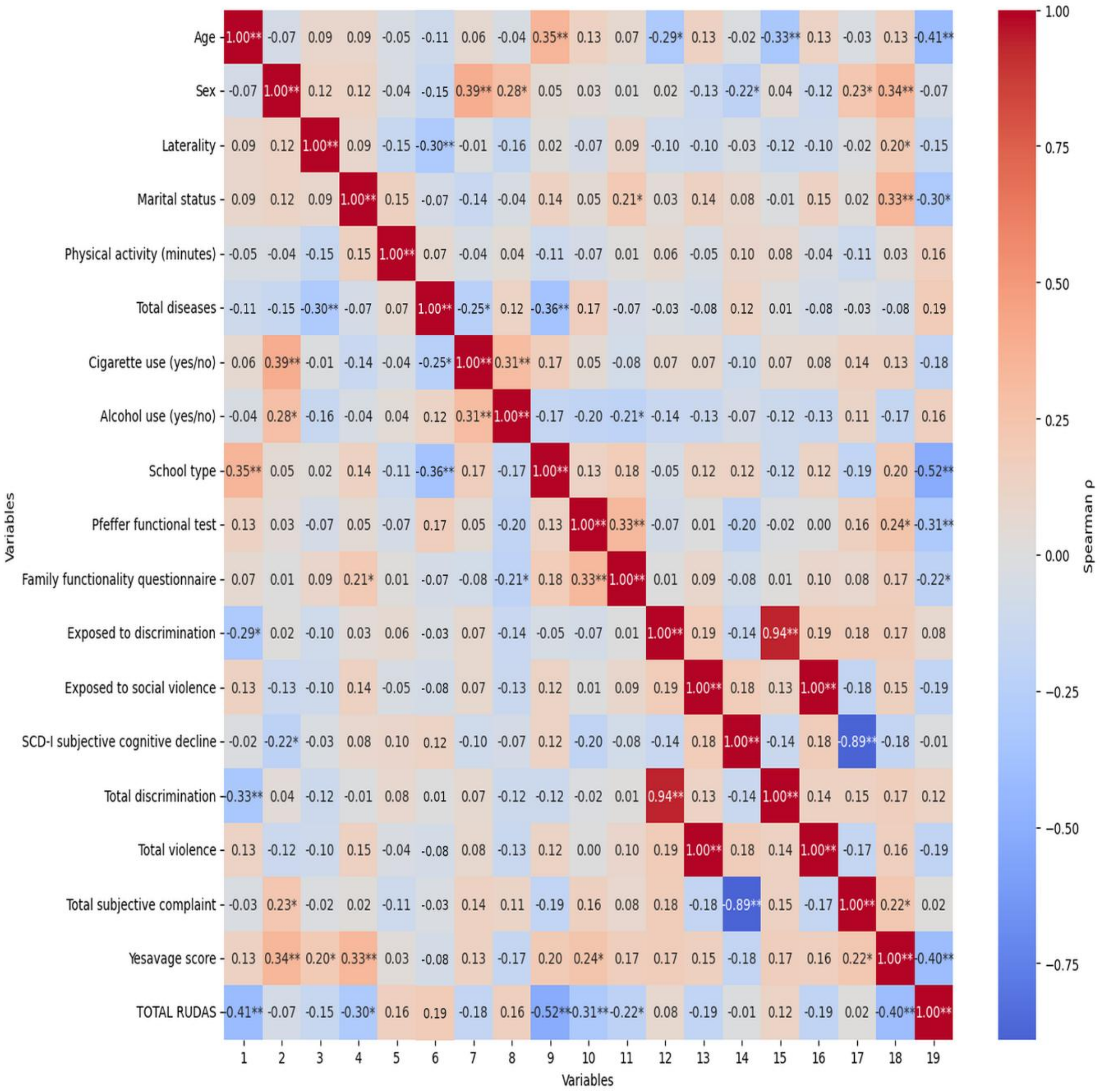

Supplementary Figure S2. Heatmap of Spearman correlation coefficients among sociodemographic, clinical, psychosocial, and cognitive variables.

Asterisks indicate statistically significant correlations (*p < 0.05; **p < 0.01)
